# Contribution of the BioFire^®^ FilmArray^®^ Meningitis/Encephalitis Panel: Assessing Antimicrobial Duration and Length of Stay

**Published:** 2019-02-26

**Authors:** Kyle Dack, Stephanie Pankow, Elizabeth Ablah, Rosey Zackula, Maha Assi

**Affiliations:** 1 University of Kansas School of Medicine-Wichita, Department of Preventive Medicine and Public Health, Wichita, KS; 2 University of Texas Health Science Center at Houston, Department of Internal Medicine, Division of Infectious Diseases, Houston, TX; 3 University of Kansas School of Medicine-Wichita, Office of Research; 4 University of Kansas School of Medicine-Wichita, Department of Internal Medicine; 5 Infectious Disease Consultants, Wichita, KS

**Keywords:** anti-infective agents, meningitis, encephalitis, length of stay

## Abstract

**Introduction:**

Traditional evaluation of meningitis includes cerebrospinal fluid (CSF) culture and gram stain to pinpoint specific causal organisms. The BioFire^®^ FilmArray^®^ Meningitis/Encephalitis (ME) Panel has been implemented as a more timely evaluation method. This study sought to assess if the BioFire^®^ ME Panel was associated with a decreased length of stay or decreased antimicrobial duration when used in the diagnosis of meningitis or encephalitis.

**Methods:**

A case, historical-control, chart review was performed on patients admitted to a regional medical center with CSF pleocytosis during Cohort 1 (the year prior to BioFire^®^ ME Panel implementation) and Cohort 2 (the year after BioFire^®^ ME Panel implementation). Length of hospital stay, duration of antimicrobials, and BioFire^®^ ME Panel result were gathered and analyzed.

**Results:**

Average length of stay for both cohorts was about four hospital days. Approximately three-fourths of all patients received antibiotic/antiviral treatment with an average of three days duration. No significant differences were observed between groups. The mean (median) duration of antimicrobials in the year prior to and after the BioFire^®^ ME Panel implementation was 3.6 (3) and 3.1 (2) days, respectively (p = 0.835). The mean (median) length of stay in the year prior to and after the BioFire^®^ ME Panel implementation was 5.8 (4) and 5.4 (4) days, respectively (p = 0.941). Among the patients admitted after the implementation of the BioFire^®^ ME Panel, 4.3 % (n = 2) had a positive bacterial result, 38.3% (n = 18) had a positive viral result, and 57.4% (n = 27) had a negative result. Of the 27 negative results, 77.8% (n = 21) were treated with antimicrobial medication.

**Conclusions:**

This study suggested there is no difference between length of stay or antimicrobial duration in presumed meningitis cases assessed with traditional methods as compared to the BioFire^®^ ME Panel.

## INTRODUCTION

Meningitis, defined as inflammation of the leptomeninges surrounding the brain and spinal cord, has a worldwide incidence of about 1.2 million cases yearly.^1^ Crucial in the evaluation of meningitis cases is the prompt retrieval of cerebrospinal fluid (CSF) via lumbar puncture to assess for organismal etiology of the infection. Evaluation of CSF includes culture and gram stain to pinpoint specific organisms causing infections. These traditional methods often take several days to render results leading to prolonged use of broad spectrum antimicrobials. Use of meningitis/encephalitis polymerase chain reaction (PCR) panels have been implemented as a supplemental organism identification method.

The BioFire^®^ FilmArray^®^ Meningitis/Encephalitis PCR Panel (BioFire^®^ ME Panel) is a multiplex PCR assay that is able to identify 14 viral, bacterial, and fungal organisms that cause meningitis or encephalitis with high diagnostic specificity and sensitivity.^2^ This diagnostic test was similar in price per patient when compared to a classic CSF culture.^3^ With the BioFire^®^ ME Panel providing a reliable diagnosis and cost effective method, the question arises about the effect the test has on course of treatment. This study assessed if the utilization of the BioFire^®^ ME Panel was associated with a decrease in length of hospital stay and duration of antimicrobials when used in the diagnosis of meningitis or encephalitis.

## METHODS

### Design

A case, historical-control study design was used to identify medical records for patients who were hospitalized for suspected meningitis. Cohort 1 included patients who were diagnosed using a cerebrospinal fluid culture and gram stain (CSF culture only: Year 1) during a hospital stay from August 7, 2015 through 2016. Cohort 2 included patients from August 8, 2016 through 2017 who were diagnosed with the BioFire^®^ ME Panel: Year 2.

### Participants

**Figure 1:**
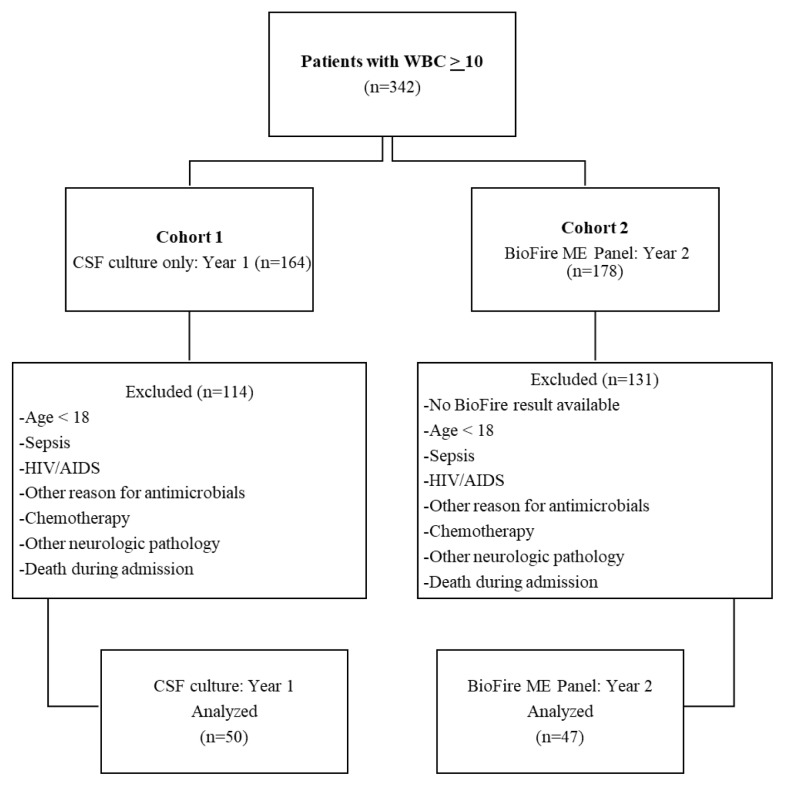
Inclusion and exclusion criteria.

Patients were included if they were 18 years or older and had white blood cell counts greater than or equal to 10. Patients were excluded if younger than 18 years of age, received antimicrobial therapy for reasons other than meningitis/encephalitis, taking chemotherapy, diagnosed with sepsis, HIV/AIDS, or other non-infectious neurological disorder or condition (Figure 1).

### Data Extraction

The OneChart EHR system was utilized to perform a chart review on patients who qualified for inclusion in the study at a regional medical center. Data regarding length of hospital stay in days (LOS), treatment with or without antimicrobials, which included duration use in days, and results from the BioFire^®^ ME Panel (positive-viral, positive-bacterial, negative, not used) were gathered and entered into a REDCap^™^ database. REDCap is a secure, web-based application that was developed specifically around HIPAA-security guidelines to house patient data. All information transmission is encrypted to protect the identity of study participants.^4^

### Analysis Plan

Data were summarized by cohort: CSF culture: Year 1 versus BioFire^®^ ME Panel: Year 2. Continuous variables included LOS and duration of antimicrobials in days. To determine appropriate statistical tests, data were evaluated for normality with the Kolmogorov-Smirnov test. As data were highly skewed, and test results were significant, variables did not pass the normality assumption. Therefore, these were summarized with medians and interquartile ranges (IQR). Cohort differences were evaluated with Mann-Whitney U tests. Categorical data included one dichotomous variable, treatment with or without antimicrobials, which were summarized by cohort using frequencies and percentages. Pearson Chi-square test was conducted to compare cohorts by treatment. Where data were sparse, exact testing procedures were utilized. Significant group differences were based on test results of p < 0.05. All statistical analyses were conducted with IBM SPSS Statistics, Version 23.

## RESULTS

During the two study periods, a total of 342 patients were reported with white blood cell counts greater than or equal to 10: 164 were in Cohort 1 (CSF culture: Year 1), while 178 were in Cohort 2 (BioFire^®^ ME Panel: Year 2; Figure 1). For Cohort 1, 50 patients met all inclusion criteria, with 114 patients being excluded. Of the 178 patients in Cohort 2, 47 met inclusion criteria with 131 patients excluded. Thus, a total of 97 patients were included in the analysis.

Table 1 illustrates group comparisons for hospital stay, antimicrobial treatment, and antibiotic/antiviral duration, along with results for BioFire^®^ ME Panel: Year 2. Groups were similar across all attributes and no significant differences were observed. Average length of stay was about four hospital days per group; approximately three-fourths of all patients received antibiotic/antiviral treatment with an average of three days duration. Among the 47 patients who were evaluated with the BioFire^®^ ME Panel, 4.3% (n = 2) had a positive bacterial result, 38.3% (n = 18) had a positive viral result, and 57.4% (n = 27) had a negative result. Among the 27 participants with a negative panel result, 77.8% (n = 21) were treated with antimicrobial medication for an average of four days (IQR: 2.0, 5.0).

Next, cohorts were compared by antimicrobial status. No significant difference was observed in hospital days between Cohort 1 and 2, regardless of treatment status. For those who were administered antimicrobials, Cohort 1 median LOS was 5.0 (IQR: 3.0, 7.0), while Cohort 2 LOS was 4.5 (IQR: 3.0, 9.0); p = 0.759. For those who did not receive antimicrobials, Cohort 1 median LOS was 2.5 days (IQR: 1.0, 4.25), and for Cohort 2 it was 1.0 days (IQR: 1.0, 5.0); p = 0.851. Although, sample size was small for each cohort without treatment, n = 14 and 11, respectively.

Further, test results showed a significant difference occurred for length of hospital stay between those who received antimicrobial treatment compared to those who did not: median hospital days with antimicrobials was 5.0 (IQR: 3.0, 7.5) versus without LOS was 2.0 (IQR: 1.0, 4.0); p < 0.001. Because data were sparse, a generalized linear model using the Gamma distribution and log link function (to account for the skewed LOS data) was conducted with a bootstrap sample of 5,000 (to account for data sparseness). Results showed that antimicrobial treatment was not a significant predictor of LOS; p = 0.223. Thus, the significant difference found above may be due to the wide variability of hospital days among participants, along with the small sample size.

## DISCUSSION

The purpose of the current study was to determine if the diagnostic utilization of the BioFire^®^ ME Panel decreased hospital length of stay and antimicrobial duration. This study suggested that utilizing this panel has little impact on the number of hospital days or treatment duration. However, data were sparse, and statistical results were inconsistent regarding length of stay with and without treatment.

These findings may be due to several factors. For example, unintentional inclusion of patients without meningitis or encephalitis (conditions would otherwise explain an elevated WBC), insufficient sample size, patient characteristics, such as sex, age, or other comorbidities that might have shed light on these results, and utilization of antimicrobials by clinicians despite negative BioFire^®^ ME Panel results. A larger scale study with similar parameters may reveal significant differences between these groups.

Of the 27 negative BioFire^®^ ME Panel results, 21 (77.8%) were continued on antimicrobial therapy despite the result. Chang et al.^5^ found similar results when assessing the BioFire^®^ ME Panel’s role in antibiotic stewardship. The current study suggested that clinicians proceed with antimicrobial treatment regardless of a negative result. This may be due to clinicians’ concerns with risk of mortality without treatment or other healthcare-related concerns.^6^

## CONCLUSIONS

The BioFire^®^ ME Panel as a diagnostic tool has the potential to target antimicrobial treatment in a timely and cost effective manner. However, evidence of its potential to decrease the use of unnecessary antimicrobials is lacking. Future investigations into antimicrobial treatment in lieu of a negative result on the BioFire^®^ ME Panel are warranted. With altered management of meningitis and encephalitis cases worked up with BioFire^®^ ME Panels and a similar study of larger caliber, the potential may exist to shorten antimicrobial duration and length of hospital stay for patients hospitalized with meningitis/encephalitis.

## Figures and Tables

**Table 1:** CSF culture versus BioFire^®^ ME Panel: hospital stay, antiviral duration, antimicrobial treatment.

		Length of Hospital Stay (days)	Treated with Antimicrobials	Antibiotic/Antiviral (days)
Diagnostic Instrument	n	Median (IQR)	n (%)	Median (IQR)
Cohort 1: CSF Culture only	50	4 (3.0, 7.0)	36 (72.0)	3 (2.5, 5.0)
Cohort 2: BioFire^®^ ME Panel	47	4 (2.0, 7.0)	36 (76.6)	3 (2.0, 5.0)
p-value		0.935*	0.648**	0.571*
BioFire^®^ ME Panel results				
Positive Bacterial	2	6 (3.0, 9.0)	2 (100.0)	6 (3.0, 9.0)
Positive Viral	18	2 (2.0, 5.0)	13 (72.2)	2 (2.0, 4.0)
Negative	27	6 (3.0, 10.0)	21 (77.8)	4 (2.0, 5.0)

IQR: Interquartile ranges

* Mann-Whitney U exact test

** Pearson chi-square exact test
